# Infiltration Pattern of Blood Monocytes into the Central Nervous System during Experimental Herpes Simplex Virus Encephalitis

**DOI:** 10.1371/journal.pone.0145773

**Published:** 2015-12-23

**Authors:** Rafik Menasria, Coraline Canivet, Jocelyne Piret, Guy Boivin

**Affiliations:** Research Center of the CHU of Quebec and Laval University, Quebec City, QC, Canada; Washington University, UNITED STATES

## Abstract

The kinetics and distribution of infiltrating blood monocytes into the central nervous system and their involvement in the cerebral immune response together with resident macrophages, namely microglia, were evaluated in experimental herpes simplex virus 1 (HSV-1) encephalitis (HSE). To distinguish microglia from blood monocyte-derived macrophages, chimeras were generated by conditioning C57BL/6 recipient mice with chemotherapy regimen followed by transplantation of bone morrow-derived cells that expressed the green fluorescent protein. Mice were infected intranasally with a sub-lethal dose of HSV-1 (1.2x10^6^ plaque forming units). Brains were harvested prior to and on days 4, 6, 8 and 10 post-infection for flow cytometry and immunohistochemistry analysis. The amounts of neutrophils (*P*<0.05) and «Ly6C^hi^» inflammatory monocytes (*P*<0.001) significantly increased in the CNS compared to non-infected controls on day 6 post-infection, which corresponded to more severe clinical signs of HSE. Levels decreased on day 8 for both leukocytes subpopulations (*P*<0.05 for inflammatory monocytes compared to non-infected controls) to reach baseline levels on day 10 following infection. The percentage of «Ly6C^low^» patrolling monocytes significantly increased (*P*<0.01) at a later time point (day 8), which correlated with the resolution phase of HSE. Histological analysis demonstrated that blood leukocytes colonized mostly the olfactory bulb and the brainstem, which corresponded to regions where HSV-1 particles were detected. Furthermore, infiltrating cells from the monocytic lineage could differentiate into activated local tissue macrophages that express the microglia marker, ionized calcium-binding adaptor molecule 1. The lack of albumin detection in the brain parenchyma of infected mice showed that the infiltration of blood leukocytes was not necessarily related to a breakdown of the blood-brain barrier but could be the result of a functional recruitment. Thus, our findings suggest that blood monocyte-derived macrophages infiltrate the central nervous system and may contribute, with resident microglia, to the innate immune response seen during experimental HSE.

## Introduction

Herpes simplex virus 1 (HSV-1) encephalitis (HSE) constitutes the most frequent sporadic and potentially fatal form of viral encephalitis in Western countries [1]. This devastating disease affects 2 to 4 individuals per million per year, although the virus infects a high percentage of the population [2]. HSE causes severe neuro-inflammation and impairment of neurological functions leading to multiple clinical features including personality changes, cognitive disorders, aphasia and seizures [2, 3]. Despite the use of intravenous acyclovir that aimed at blocking virus replication, the mortality rate associated with HSE is still high (i.e., 20–30%), with the majority of surviving patients developing severe neurological sequelae [2, 4]. The mechanisms underlying the pathogenesis of HSV-1 infection of the central nervous system (CNS) have not been completely elucidated. It is believed that the high mortality rate attributable to HSE could involve both virally- and immune-induced brain damages [5–9].

The control of HSV-1 infection in the CNS is dependent on the cellular immune response including local tissue macrophages, namely microglia [10–12]. It has been demonstrated that microglia can control virus replication in the brain by producing inflammatory cytokines and chemokines such as interleukin (IL)-6, IL-1β, type I interferons (IFN-I), C-X-C motif ligand 10 (CXCL10), C-C motif ligand 2 (CCL2) and CCL5 [13, 14]. However, animal studies revealed that this response may not be sufficient to protect from HSV-1 infection in the CNS [15]. In fact, intranasal inoculation of HSV-1 to susceptible BALB/c mice led to vigorous and yet non-protective microglial activation. Importantly, flow cytometry analysis suggested that, in addition to resident microglial activation, infiltration of peripheral monocytes and neutrophils into the CNS could occur and worsen HSE outcome by amplifying the cerebral inflammatory state, thereby increasing mortality [15, 16]. However, the precise infiltration pattern of different blood leukocytes subpopulations and their distribution in the CNS during experimental HSE has not been well defined [7, 17–19]. In fact, monocyte-derived macrophages and activated microglia cannot be reliably discriminated on brain sections based on their morphology, cell surface markers or localization. It is thus challenging to precisely determine the kinetics, brain localization and the relative contribution of these cells to mount a cerebral immune response [20].

Furthermore, murine blood monocytes can be classified into two principal subpopulations: inflammatory «Ly6C^hi^/CX3CR1^low^/CCR2^+^» and patrolling «Ly6C^low^/CX3CR1^hi^/CCR2^−^» monocytes [21, 22]. These two subsets of monocytes express distinct chemokine receptors and adhesion molecules which reflect their respective functions and migratory patterns [23]. The inflammatory monocytes infiltrate inflamed tissues in a CCR2-dependent manner and exert pro-inflammatory, phagocytic and proteolytic functions essential for damaged tissue digestion and debris removal [24–26]. In contrast, patrolling monocytes exert anti-inflammatory functions and are involved in tissue regeneration, growth, angiogenesis and matrix deposition [26–30]. It has been demonstrated that both subtypes of monocytes as well as neutrophils can gain access to the CNS following cerebral insults and engage effector functions including antigen presentation and production of pro-inflammatory mediators, which allow antigens containment and clearance [16, 31–35]. However, during HSE, the inflammatory response mediated by both microglia and infiltrating leukocytes can become uncontrolled and worsen the outcome of the disease.

In order to investigate the infiltration pattern of blood monocytes during HSE, our group had previously conducted a tracing study in which chimeric C57BL/6 mice were generated using whole body irradiation followed by transplantation of bone marrow-derived cells expressing the green fluorescent protein (GFP). We showed that GFP^+^ blood-derived leukocytes were detected in the CNS following intranasal inoculation of HSV-1. However, this myeloablative process also induced non-specific blood cells infiltration in the CNS of non-infected mice. This irradiation-related infiltration made it difficult to distinguish newly infiltrating blood-derived leukocytes induced by HSV-1 infection from resident microglia [19]. In addition, this experiment did not evaluate the kinetics and distribution of infiltrating blood leucocyte subpopulations in the CNS.

Here, we generated chimeras using C57BL/6 recipient mice conditioned with a myeloablative chemotherapy regimen consisting of the alkylating agent busulfan and the immunosuppressant cyclophosphamide. It has been previously demonstrated that such myeloablation procedure followed by transplantation of bone marrow cells derived from GFP^+^ transgenic mice was sufficient to induce a strong chimerism both in the blood and hematopoietic organs without affecting brain integrity [36]. Using this chimeric mouse model, we investigated the brain localization of blood leukocytes as well as the infiltration kinetics of neutrophils, inflammatory and patrolling monocytes into the CNS during HSE.

## Materials and Methods

### Animals

Wild-type (WT) male C57BL/6J mice and hemizygous transgenic male C57BL/6J mice expressing GFP (GFP^+/-^) under control of the chicken β-actin promoter and the cytomegalovirus enhancer [B6-Tg (CAG-EGFP) 1 Osb/J] were obtained from Jackson Laboratory (Bar Harbor, ME). All animals were used in accordance to the Canadian Council on Animal Care guidelines and all protocols were approved by the Animal Care Ethics Committee of Laval University.

### Myeloablative chemotherapy conditioning and bone marrow transplantation

Eight to nine week-old male C57BL/6J recipient mice were housed in autoclaved cages and received irradiated mouse chaw. Mice were treated with 0.2 mg trimethoprim and 1 mg sulfamethoxazole (SEPTRA, GlaxoSmithKline, Mississauga, Ontario, Canada) per mL of sterile water *ad libitum* starting 1 week before and maintained for 2 weeks after transplantation. The chemotherapy regimen consisted of twice daily injections of 10 mg/kg of busulfan (Otsuka, St-Laurent, Quebec, Canada) for 4 days (for a total of 80 mg/kg) followed by once daily injection of 100 mg/kg of cyclophosphamide (Baxter, Mississauga, Ontario, Canada) for 2 days (for a total of 200 mg/kg). All injections were performed intraperitoneally (i.p.) in a total volume of 150 μL.

Bone marrow cells from age- and sex-matched GFP^+/-^ mice were aseptically harvested by flushing the femurs and tibias with Dulbecco phosphate-buffered saline (DPBS) containing 1g/L glucose and 36 mg/L sodium pyruvate and supplemented with 2% fetal bovine serum (FBS; Wisent, St-Bruno, Quebec, Canada). Cells were filtered through a 40-μm cell strainer (BD Biosciences, San Jose, CA), washed three times in FBS-free DPBS (by centrifugation at 300 × g for 5 min) and counted with a hemacytometer. Recovered cells (adjusted to 1.5×10^7^ in 200 μL DPBS) were then injected in the tail vein of recipient mice 24 h following the chemotherapy regimen. To prevent chemotherapy-induced dehydration, mice received once daily injection of 1 mL saline subcutaneously.

### Flow cytometry procedures for evaluation of chimerism in the blood

Blood samples (~120 μL) were withdrawn from the facial vein of C57BL/6 (WT) recipient mice 8 weeks after transplantation with GFP^+^ bone marrow cells (GFP^+/-^→WT) (n = 15) as well as of GFP^+/-^ (n = 9) and WT (n = 6) animals. Samples were quickly collected in EDTA-coated tubes (Starstedt, Montreal, Quebec, Canada) to prevent coagulation. A volume of 35 μL of DPBS without Ca^2+^ and Mg^2+^ (Sigma-Aldrich, St Louis, MO) was added to 65 μL of blood and incubated for 20 min on ice with purified rat anti-mouse CD16/CD32 antibody diluted 1:100 (clone 2.4G2; BD Biosciences) to block non-specific binding of IgGs to Fc receptors. Samples were washed and resuspended in 100 μL of DPBS after being centrifuged at 300 x g for 10 min. Cell suspensions were then labeled with the following rat anti-mouse antibodies for 40 min at 4°C: PE-Cy5-CD45 (clone 30-F11; BD Biosciences), APC-CD115 (clone AF598; eBioscience, San Diego, CA), PE-Cy7-CD11b (clone M1/70; eBioscience), V450-Ly6C (clone AL21; BD Biosciences) and PE-Ly6G (clone 1A8; BD Pharmingen, San Jose, CA). Red blood cells were lysed with BD Pharm Lyse™ (BD Biosciences) during 30 min at room temperature, and the recovered leukocytes were washed and resuspended in DPBS for analysis. Flow cytometry analysis and data acquisition were performed using a BD SORP LSR II and the BD FACSDiva software, respectively.

### Infection of mice with HSV-1

Eight weeks post-transplantation, chimeric mice were infected intranasally with a sub-lethal dose consisting in 1.2x10^6^ plaque forming units (PFU) of HSV-1 clinical strain H25 (grown and passaged in Vero cells) in 20 μL minimal essential medium as described elsewhere [37].

### Flow cytometry procedures for brain leukocytes

Prior to and on days 4, 6, 8 and 10 post-infection, mice (5 animals for each time point) were deeply anesthetized with an i.p. injection of a mixture of ketamine hydrochloride (Bioniche Animal Health, Belleville, Ontario, Canada) and xylazine (Bimeda, Cambridge, Ontario, Canada) and then perfused intracardially with ice-cold DPBS without Ca^2+^ and Mg^2+^. Brains were extracted and immediately homogenized with a plunger in 20 mL of DPBS supplemented with 0.077 mg of Liberase TL (Roche Diagnostics, Mannheim, Germany) and incubated for 1 h at 37°C. Brain homogenates were then filtered through a 70-μm cell strainer (BD Biosciences). The cell suspension was centrifuged at 300 x g for 10 min at room temperature. The supernatant was aspirated and cells were gently resuspended in 7 mL of 37% Percoll (GE Healthcare, Uppsala, Sweden). The cell suspension was underlaid beneath 80% Percoll and centrifuged at 600 x g for 40 min with slow acceleration and deceleration rates. The cell ring at the interphase was collected and mixed thoroughly with DPBS containing 2% FBS. Cells were then centrifuged at 300 x g for 10 min and washed twice with DPBS plus 2% FBS. Cells were first incubated on ice for 35 min with purified rat anti–mouse CD16/CD32 (Mouse Fc Block; clone 2.4G2; BD Biosciences). After a washing step, cells were incubated for 40 min on ice with the same pool of secondary antibodies described above except APC-CD115. Flow cytometry analysis and data acquisition were performed using a BD SORP LSR II and the BD FACSDiva software, respectively.

### Immunohistochemistry analysis

For immunohistochemistry studies, 5 to 6 mice were sacrificed prior to and on days 4, 6, 8 and 10 post-infection by intracardiac perfusion with cold 0.9% saline followed by a 4% paraformaldehyde solution (pH 7.4) at 4°C. Brains were removed, post-fixed in a paraformaldehyde solution for 24 h, and placed in a 15% sucrose solution prepared in 4% paraformaldehyde at 4°C at least for 48 h. Brains were cut in 25-μm coronal sections on dry ice with a microtome (Reichert-Jung, Cambridge Instruments Company, Deerfield, IL). Sections were collected in a cold cryoprotectant solution (0.05 M sodium phosphate buffer at pH 7.3 containing 30% ethylene glycol and 20% glycerol) and stored at −20°C. Free-floating sections were washed 3 times for 15 min in potassium PBS (KPBS) and incubated for 30 min in KPBS containing 4% goat (Cederlane Laboratories, Hornby, Ontario, Canada) or chicken (Rockland Immunochemicals, Limerick, PA) serum, 1% BSA and 0.4% Triton X-100 (both from Sigma-Aldrich). Sections were incubated overnight with primary antibodies in the same buffer solution diluted 1/2 at 4°C. The list of primary and secondary antibodies used and their dilution are given in Table 1. Following incubation, sections were rinsed 3 times for 15 min in KPBS, followed by 90 min incubation with fluorochrome-conjugated secondary antibodies at room temperature. Finally, nuclear staining was performed using DAPI (diluted to 0.0002% for 10 min; Molecular Probes, Eugene, OR). Sections were then rinsed 3 times for 10 min in KPBS before being mounted onto SuperFrost slides (Fisher Scientific, Nepean, Ontario, Canada) and coverslipped with Fluoromount-G (SouthernBiotech, Birmingham, AL). An ischemic tissue used as a positive control for the evaluation of blood-brain barrier integrity was kindly provided by Dr Serge Rivest (Laval University, Quebec, Canada) [38]. Standard and confocal fluorescence microscopy images were respectively captured using a Nikon Eclipse 80i microscope (Nikon, Melville, NY) equipped with a digital camera (QImaging, Surrey, British Columbia, Canada) and a Confocal Quorum WaveFX spinning disk confocal microscope (Quorum Technologies, Guelph, Ontario, Canada) equipped with a Hamamatsu ImageEM camera (Hamamatsu Corporation, Bridgewater, NJ). Images were acquired using the Volocity 4 software (Perkin Elmer, Waltham, MA). Regions of the CNS were identified based on the mouse brain atlas (http://atlas.brain-map.org).

**Table 1:** Primary and secondary antibodies used for immunohistochemistry analysis.

Target	Immunogen	Manufacturer	Conjugated	Dilution
Green fluorescent protein (GFP)	Fusion protein identical to full-length GFP	Invitrogen	No	1/1000
Iba1	Synthetic peptide C-TGPPAKKAISELP corresponding to the C-terminal portion of Iba1	Wako Chemicals	No	1/3000
HSV-1	Infected-cell proteins (ICPs) and late structural (virion) antigens	ABD Serotec	No	1/1200
CD68	Mouse macrosialin, a heavily glycosylated transmembrane protein and murine homolog of human CD68	ABD Serotec	No	1/1000
MHC II	Polymorphic determinant shared by the I-A[b], I-A[d], I-A[q], I-E[d], I-E[k] and cells from mice of the H-2[p] and H-2[r] haplotypes	BD Pharmingen	No	1/1000
Albumin	Not defined	Jackson Immunoresearch	No	1/1000
Goat IgG	Purified from chicken serum	Invitrogen	Alexa 488	1/1000
Rabbit IgG	Purified from chicken serum	Invitrogen	Alexa 594	1/1000
Rabbit IgG	Purified from goat serum	Jackson Immunoresearch	Cy5	1/2000
Rat IgG	Purified from goat serum	Invitrogen	Alexa 568	1/1000
Rat IgG	Purified from goat serum	Invitrogen	Cy5	1/2000

### Statistical analyses

Body weight changes and flow cytometry data were analyzed by a one-way analysis of variance (ANOVA) with Tukey's multiple comparison post-test. All statistical analyses were carried out using the GraphPad Prism software program, version 5.00 (GraphPad Software, San Diego, CA). A *P* value of less than 0.05 was considered statistically significant.

## Results

### Chemotherapy regimen induces a strong peripheral chimerism following bone marrow transplantation

**Fig 1:**
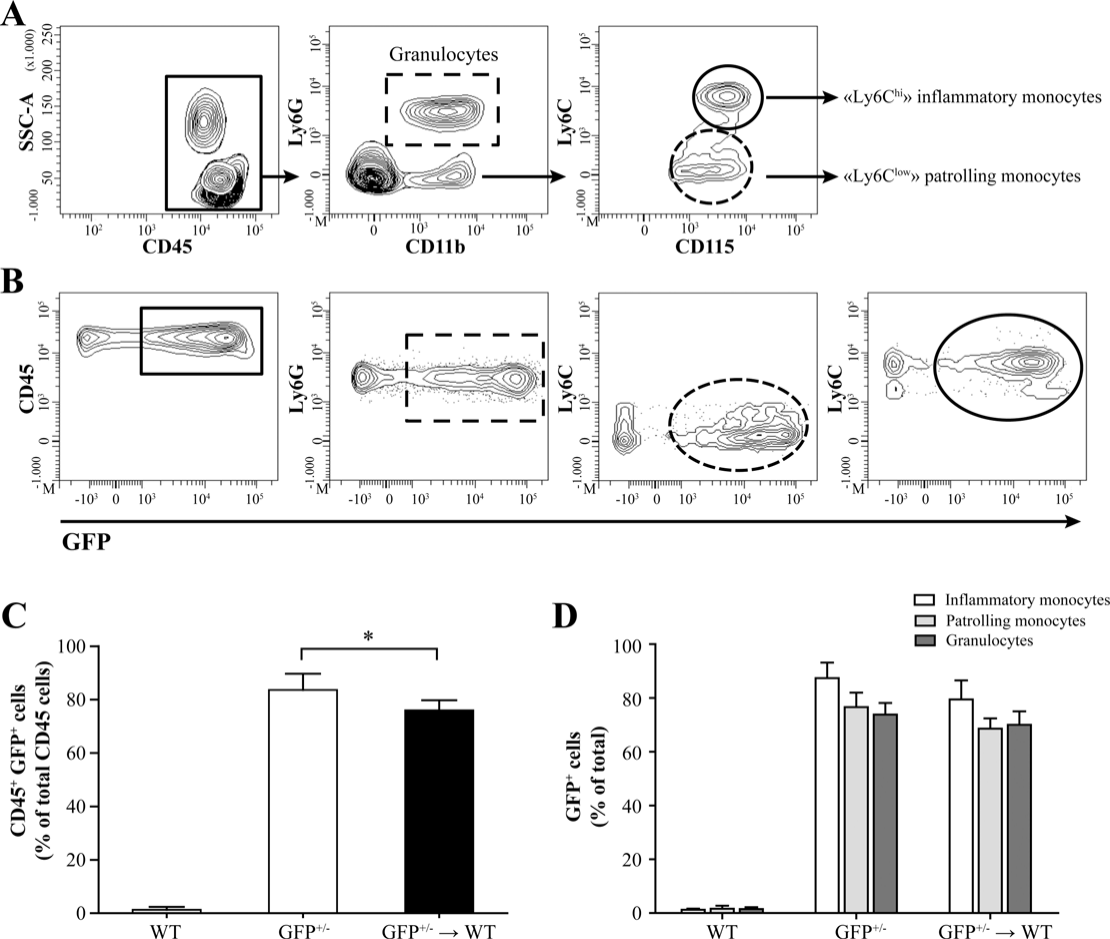
Chemotherapy results in a high blood cells chimerism in GFP^+/-^→ WT mice. Blood chimerism was assessed by flow cytometry 8 weeks following transplantation of GFP^+/-^ bone marrow-derived cells in wild-type (WT) C57BL/6 recipient mice conditioned with busulfan and cyclophosphamide (n = 15) and compared with WT (n = 6) and GFP^+/-^ mice (n = 9). (A) Representative flow cytometry contour plots showing the gating strategy for blood leukocytes (i.e., CD45^+^) (continuous rectangle), granulocytes (CD45^+^/CD11b^+^/Ly6G^+^) (dashed rectangle), inflammatory monocytes (CD45^+^/CD11b^+^/CD115^+^/Ly6C^hi^) (continuous circle) and patrolling monocytes (CD45^+^/CD11b^+^/CD115^+^/Ly6C^low^) (dashed circle). (B) Representative flow cytometry contour plots illustrating the strategy used to determine GFP expression in each of these leukocyte populations. (C) Histogram illustrating the percentage of CD45^+^/GFP^+^ cells compared to total leukocytes (CD45^+^) in WT, GFP^+/-^ and GFP^+/-^→WT mice and (D) histogram showing the evaluation of GFP expression in the different blood cell populations of the myeloid lineage in all three groups of mice. Results are from two independent experiments. Statistical analyses were performed using a one-way analysis of variance (ANOVA) with Tukey's multiple comparison post-test. *, *P*≤0.05 compared to GFP^+/-^ mice.

Chimeras were generated by subjecting WT C57BL/6 mice to a myeloablative chemotherapy regimen and transplantation of bone marrow-derived cells harvested from GFP^+/-^ transgenic donor mice (GFP^+/-^→WT). To evaluate the efficiency of the chemotherapy treatment and the replenishment of the hematopoietic system of recipient mice by newly transplanted cells, the levels of GFP-labeled peripheral blood cells were analyzed using flow cytometry eight weeks after transplantation in GFP^+/-^→WT compared to GFP^+/-^ and WT mice. The expression of the cell surface marker CD45 was first utilized to target blood leukocytes. The granulocytes marker Ly6G was then used to discriminate neutrophils (CD45^+^/CD11b^+^/Ly6G^+^) from other leukocytes. Finally, monocytes were differentiated according to CD115 and Ly6C expression level into Ly6C^hi^ and Ly6C^low^ cells which were associated with inflammatory (CD45^+^/CD11b^+^/CD115^+^/Ly6C^hi^) and patrolling (CD45^+^/CD11b^+^/CD115^+^/Ly6C^low^) monocytes, respectively (Fig 1A). The expression of GFP was evaluated in each of these leukocyte subtypes (Fig 1B). As expected, a high percentage of GFP^+^ leukocytes were detected in GFP^+/-^ mice (mean±SD; 83.6%±6.1) whereas it was much lower or null in WT (1.3%±1.1) (Fig 1C). Importantly, the percentage of GFP-labelled cells in the blood of GFP^+/-^→WT mice (76.0%±4.8) was only slightly lower than that of GFP^+/-^ animals indicating that blood leukocytes in recipient mice were mostly from donor origin. Detailed evaluation of GFP expression in leukocytes of the myeloid lineage in GFP^+/-^→WT mice revealed that high chimerisms in inflammatory monocytes (79.4%±7.1), patrolling monocytes (68.8%±3.8) and granulocytes (70.0%±5.0) were non-significantly different compared to those of GFP^+/-^ donor mice (87.4%±5.8, 76.4%±5.4 and 73.8%±4.3, respectively) (Fig 1D). Overall, these data suggest that the recipient’s hematopoietic system was almost entirely replenished with donors’ bone marrow cells.

### Both monocyte subsets and neutrophils infiltrate the CNS following HSV-1 infection

**Fig 2:**
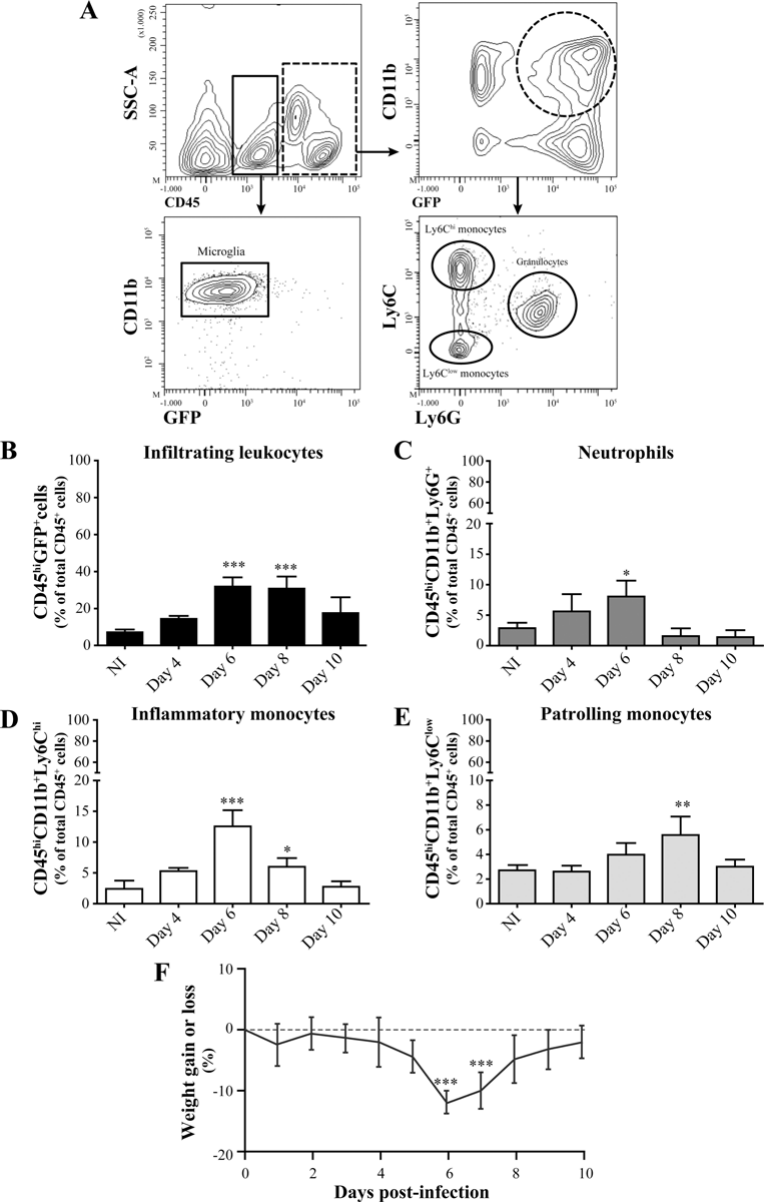
Kinetics of infiltrating blood myeloid leukocytes in the CNS during HSE. Brain leukocytes of GFP^+/-^→WT mice were analysed by flow cytometry prior to and on days 4, 6, 8 and 10 following intranasal infection with 1.2x10^6^ PFU of HSV-1. (A) Flow cytometry contour plots illustrating the gating strategy used for brain leukocytes differentiation in mice sacrificed on day 6 following infection. CD45 marker was used to discriminate microglia (i.e., CD45^low^) (continuous rectangle) from infiltrating leukocytes (i.e., CD45^hi^) (dashed rectangle). Among CD45^hi^/CD11b^+^/GFP^+^ infiltrating myeloid cells (dashed circle), neutrophils were selected based on the expression of the granulocyte marker Ly6G whereas monocyte subsets were discriminated according to Ly6C expression level into Ly6C^hi^ inflammatory monocytes and Ly6C^low^ patrolling monocytes. (B) Infiltrating leukocytes, (C) neutrophils, (D) inflammatory monocytes and (E) patrolling monocytes percentages with respect to total brain leukocytes were evaluated during HSE. Histograms represent data obtained with n = 5 to 6 mice per time point. (F) Percentage of body weight changes in GFP^+/-^→WT mice following infection with HSV-1 (n = 9 mice). Statistical analyses were performed using a one-way analysis of variance (ANOVA) with Tukey's multiple comparison post-test. *, *P*<0.05; **, *P*<0.01; ***, *P*<0.001 compared to non-infected group.

To evaluate the infiltration of blood leukocytes into the CNS in our mouse model of HSE, GFP^+/-^→WT mice were infected with a sub-lethal inoculum (1.2x10^6^ PFU) of HSV-1 by the intranasal route. The amounts of the different leukocyte populations in the brain were then analyzed by flow cytometry prior to and on days 4, 6, 8 and 10 following the infection. These different time points have been selected based on the severity of clinical signs (i.e., weight loss, ruffled fur, swollen forehead and excitability) that reaches a peak on day 6 post-infection in our mouse model of HSE [19, 37, 39]. In our study, GFP and CD45 markers were first used to distinguish CD45^low^/CD11b^+^/GFP^-^ from CD45^hi^/CD11b^+^/GFP^+^ cells which were attributed to resident microglia and infiltrating leukocytes, respectively (Fig 2A). Neutrophils, inflammatory and patrolling monocytes were discriminated from other infiltrating leukocytes using their specific cell surface markers; i.e., CD45^hi^/CD11b^+^/GFP^+^/Ly6G^+^, CD45^hi^/CD11b^+^/GFP^+^/Ly6C^hi^ and CD45^hi^/CD11b^+^/GFP^+^/Ly6C^low^, respectively (Fig 2A). Analysis of GFP expression revealed that almost all «CD45^low^» resident microglia (99.2%) were GFP^-^ prior to infection which suggested a recipient origin and excluded a non-specific engraftment of blood monocytes in brain of mice following myeloablation and transplantation (data not shown). There was also no expression of GFP in «CD45^low^» microglia following infection (98.9%) suggesting that bloodstream leukocytes did not participate to the replenishment of this cell population during HSE (Fig 2A). Prior to infection, 7.3% of all brain leukocytes (i.e., CD45^+^) expressed CD45^hi^/GFP^+^ markers and were attributed to infiltrating peripheral immune cells. This percentage significantly increased on days 6 (31.9%; *P*<0.001) and 8 (30.9%; *P*<0.001) and then decreased on day 10 (17.6%) following infection (Fig 2B). These data suggest that immune cells infiltrated the brain mainly on day 6 following infection. The infiltration of neutrophils into the CNS increased from day 4 (5.6%) to day 6 (8.5%; *P*<0.05) post-infection compared to non-infected controls (2.9%). Thereafter, their amount decreased to baseline levels on days 8 and 10 post-infection (Fig 2C). The level of «Ly6C^hi^» inflammatory monocytes was slightly increased on day 4 (5.3%) and was significantly higher on day 6 following infection (12.5%; *P*<0.001) compared to non-infected mice (2.4%). Their amount decreased on day 8 (5.9%; *P*<0.05) to finally reach baseline levels on day 10 following infection (2.8%) (Fig 2D). Interestingly, the percentage of «Ly6C^low^» patrolling monocytes, which are associated with the resolution phase of the inflammatory response, slowly increased to reach a peak on day 8 following infection (5.5%; *P*<0.01) compared to controls (2.7%) (Fig 2E). Overall, our data suggest that neutrophils and inflammatory monocytes infiltrate the CNS mainly on day 6 following infection, which corresponded to the most severe clinical signs of HSE as indicated by the greater weight loss at this time point (Fig 2F) whereas patrolling monocytes peak at a later time (day 8). Considering the fact that the GFP marker was used to identify infiltrating cells, it is important to note that the percentages of the different cell populations in the CNS were underestimated since GFP^+^ blood leukocytes represented approximately 80% of the total CD45^+^ cells (Fig 1C).

### Infiltrating leukocytes are mainly located in the olfactory bulb and the brainstem of infected chimeric C57BL/6 mice

**Fig 3:**
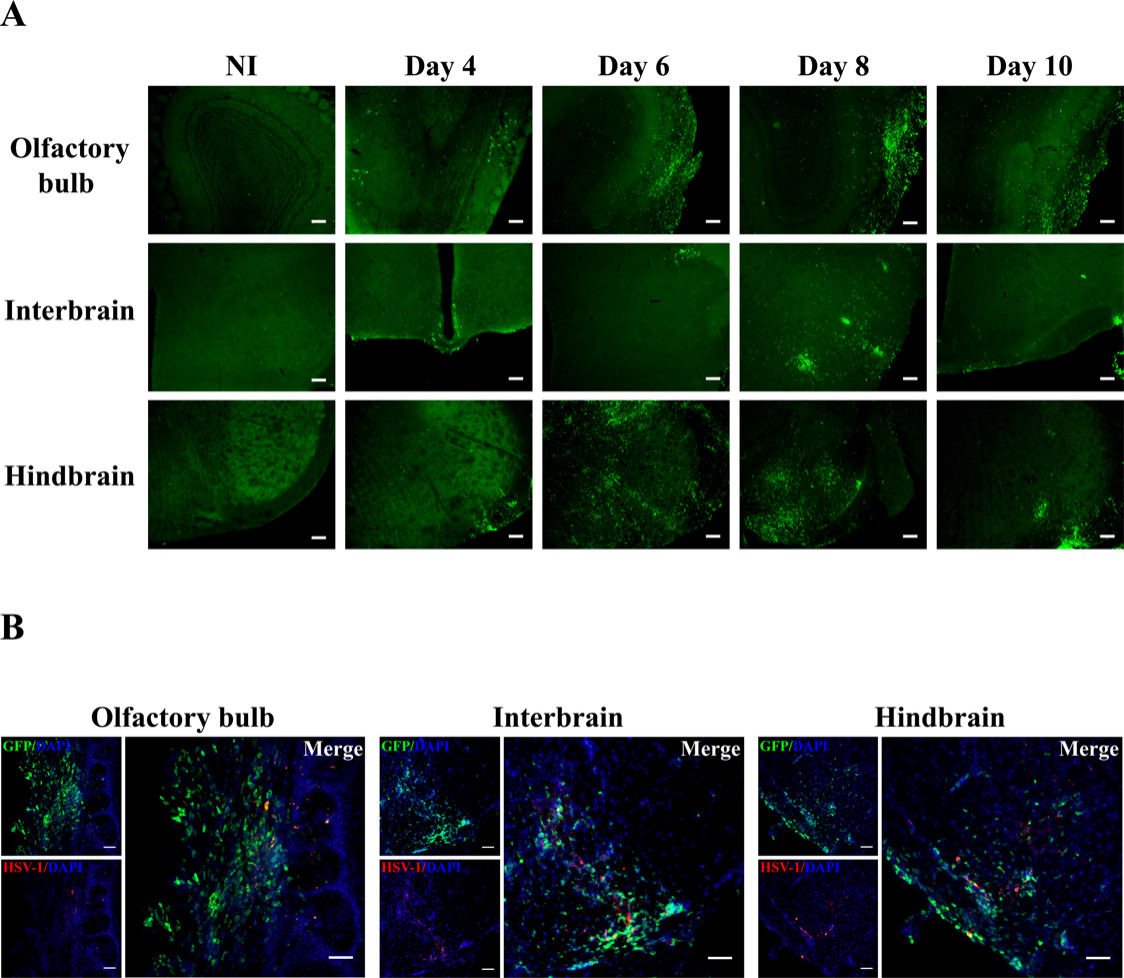
Intranasal infection with HSV-1 induces blood leukocytes infiltration in the CNS of chimeric C57BL/6 mice. Representative micrographs illustrating the localization of GFP^+^ infiltrating cells in the olfactory bulb, the interbrain and the hindbrain of GFP^+/-^→WT mice. Sixteen week-old chimeric mice were infected with HSV-1 by the intranasal route and sacrificed prior to (negative control) and on days 4, 6, 8 and 10 post-infection (5 mice per group). (A) Brain slices of 25-μm thick were processed for immunohistochemistry staining with a primary polyclonal goat anti-GFP and a secondary Alexa 488-conjugated chicken anti-goat antibodies (green). In non-infected mice (NI), no GFP^+^ cells could be found in the brain parenchyma. Following infection, peripheral leukocytes infiltrated the CNS and could be detectable mainly in the olfactory bulb, the interbrain and the hindbrain. Scale bar 100 μm. (B) Representative micrographs illustrating the localization of HSV-1 particles in different regions of the brains of GFP^+/-^→WT mice on day 6 post-infection. Brain slices were processed for immunohistochemistry analysis with a primary polyclonal rabbit anti-HSV-1 antibody and a secondary Alexa 594-conjugated goat anti-rabbit antibody (red), followed by nuclear staining with DAPI (blue). Viral antigens were detected in the olfactory bulb, the interbrain and the hindbrain. Scale bar 50 μm.

To evaluate the kinetics and distribution of leukocytes infiltration into the CNS during HSE, GFP^+/-^→WT mice were infected intranasally with HSV-1 and sacrificed prior to and on days 4, 6, 8 and 10 following infection to obtain brain tissue sections. Our results showed that no GFP-expressing cells could be detected in the CNS parenchyma of non-infected chimeric mice in any regions of the brain (Fig 3A). GFP^+^ cells could only be found in areas not protected by the blood-brain barrier (BBB) such as the choroid plexus (data not shown). On day 4 post-infection, GFP^+^ cells were detected in the olfactory bulb (OB) and the brainstem. More specifically in the brainstem, GFP^+^ cells could be found in the interbrain (which corresponds to the thalamic and hypothalamic areas) and the hindbrain (which corresponds to the pons (data not shown) and medulla) (Fig 3A). In agreement with flow cytometry analysis (Fig 2B), an important infiltration of blood leukocytes was observed on day 6 following infection. These cells were mainly located in the OB, and to a lesser extent in the interbrain. Importantly, blood-derived leukocytes were also detected in different regions of the hindbrain including the pons (not shown) and the medulla (Fig 3A) as well as in the cerebellum (not shown). These cells were localized in the same anatomical areas on days 8 and 10 following infection (Fig 3A). Interestingly, infiltrating leukocytes mostly colonized brain regions where HSV-1 particles were found. Indeed, virus particles were detectable in the OB, interbrain and hindbrain on days 6 (Fig 3B) and 8 (data not shown) whereas they were no longer visible on day 10 following infection (data not shown). These data suggest that blood leukocytes are recruited into the CNS during HSE and reach specific areas which closely correspond to HSV-1-infected regions.

### Blood monocytes can infiltrate the CNS without a breakdown of the blood-brain barrier

**Fig 4:**
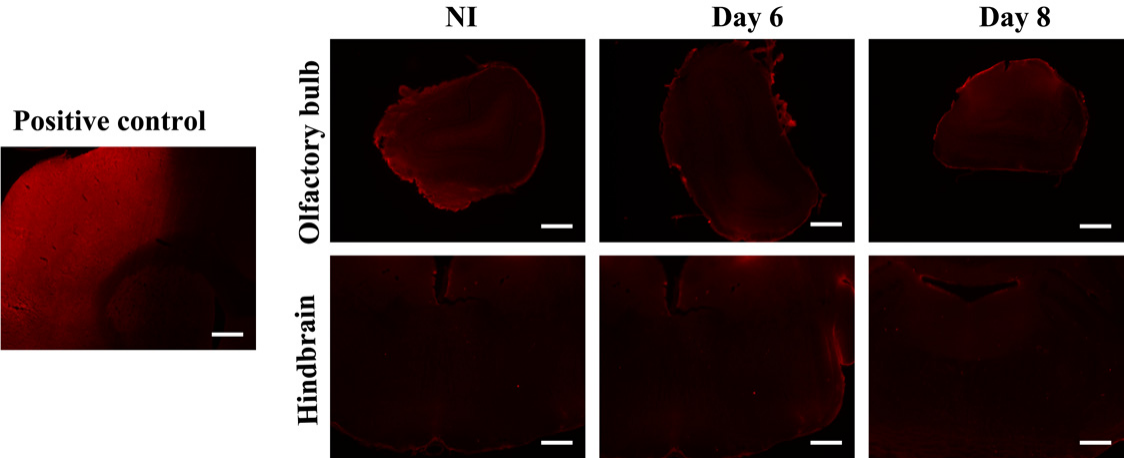
Evaluation of blood-brain barrier integrity in chimeric C57BL/6 mice following HSV-1 infection. Micrographs illustrating the detection of albumin in the brain parenchyma of non-infected (NI) animals and mice sacrificed on days 6 and 8 following HSV-1 infection. Brain slices were processed for immunohistochemistry with primary goat anti-albumin and secondary Alexa 594-conjugated chicken anti-goat (red) antibodies. Pictures selected showed no immuno-reactivity for albumin in non-infected mice in the olfactory bulb and hindbrain. Positive control corresponds to a slice of an ischemic brain tissue obtained in a mouse model of stroke. Scale bar 400 μm.

In order to determine whether GFP^+^ peripheral cells infiltration resulted from a functional recruitment or alteration of the BBB surrounding CNS vasculature, brain sections were stained for the blood protein albumin. Images depicted in Fig 4 represent brain slices of the OB and the hindbrain where GFP^+^ cells were observed (Fig 3A). No immunostaining for albumin was detected in the OB and the hindbrain (Fig 4) or any other regions of the brain (data not shown) of non-infected mice. Furthermore, there was no immuno-reactivity for albumin in the brain parenchyma of mice sacrificed on days 4 (data not shown), 6, 8 (Fig 4) and 10 (data not shown) following infection when compared to the positive control (an ischemic tissue obtained in a mouse model of stroke [38] which was, as expected, immuno-reactive for albumin), suggesting that leukocytes infiltration was not related to a breakdown of the BBB.

### Once in the CNS, infiltrating monocytes express the resident microglia marker Iba1

**Fig 5:**
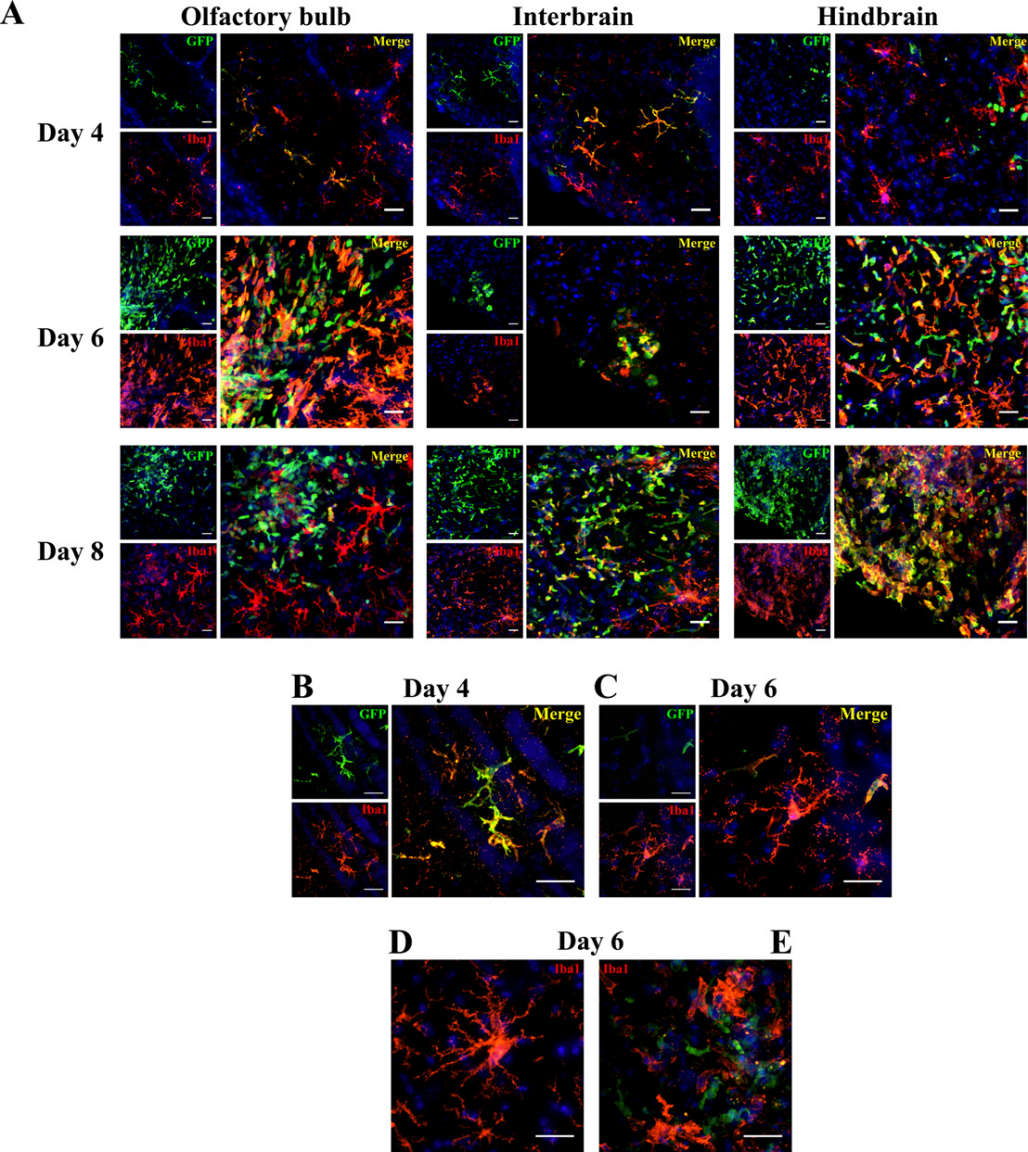
Blood-derived GFP^+^ monocytes that infiltrate the brain parenchyma following HSV-1 infection express the microglia marker Iba1. GFP^+/-^→WT mice were infected with HSV-1 via the intranasal route and sacrificed prior to and on days 4, 6, 8 and 10 following infection. (A) The location and phenotype of engrafted GFP^+^ cells were assessed in brain sections by double immunostaining with goat anti-GFP and rabbit anti-Iba1 followed by secondary antibodies, Alexa 488-conjugated chicken anti-goat (green) and Alexa 594-conjugated chicken anti-rabbit (red), respectively. Nuclear staining with DAPI is shown in blue. Scale bar 25 μm. Higher magnification of brain sections illustrating the ramified (B) and amoeboid (C) forms of infiltrating monocytes (GFP^+^) that expressed the microglia marker Iba1 on days 4 and 6 post-infection, respectively. Panels (D) and (E) show resident microglia (Iba1^+^/GFP^-^) with ramified and amoeboid morphologies related to resting and activated states, respectively, on day 6 following infection. Scale bar 25 μm.

To further determine the fate of infiltrating monocytes in the CNS during HSE, the marker Iba1, which is specifically expressed by brain macrophages/microglia, was used to distinguish GFP^+^ cells of the monocytic lineage that differentiated into resident macrophages from other leukocyte subpopulations. Our results showed that ramified GFP^+^ cells found on day 4 following infection in the OB and the interbrain (Fig 5A) also expressed Iba1, indicating that they differentiated into cells resembling resident microglia. In contrast, at the same time point, GFP^+^ cells located in the hindbrain were not labelled with Iba1 suggesting that they may belong to other cell types. In addition to the ramified cells observed on day 4, amoeboid Iba1^+^/GFP^+^ cells were found in the OB, the interbrain and the hindbrain on days 6, 8 (Fig 5A) and 10 (data not shown) post-infection. It has been demonstrated that ramified microglia are able to change their morphology to reactive or amoeboid form in response to a variety of CNS insults such as virus invasion [40]. In line with these studies, higher magnification of brain slices showed that infiltrating macrophages (Iba1^+^/GFP^+^) could adopt a ramified (Fig 5B) or an amoeboid form (Fig 5C) on days 4 and 6 post-infection, respectively. Furthermore, Iba1^+^/GFP^-^ resident microglia could also adopt the ramified form (observed in either non-infected mice (data not shown) or on day 6 (Fig 5D)) as well as the amoeboid morphology (Fig 5E), which was only observed following infection. Overall, our results indicated that cells of the monocytic lineage have the ability to infiltrate the CNS during HSE and differentiate into macrophages adopting a microglia profile (Iba1^+^).

### Both CNS resident microglia and blood-derived macrophages are involved in immune functions during HSE

**Fig 6:**
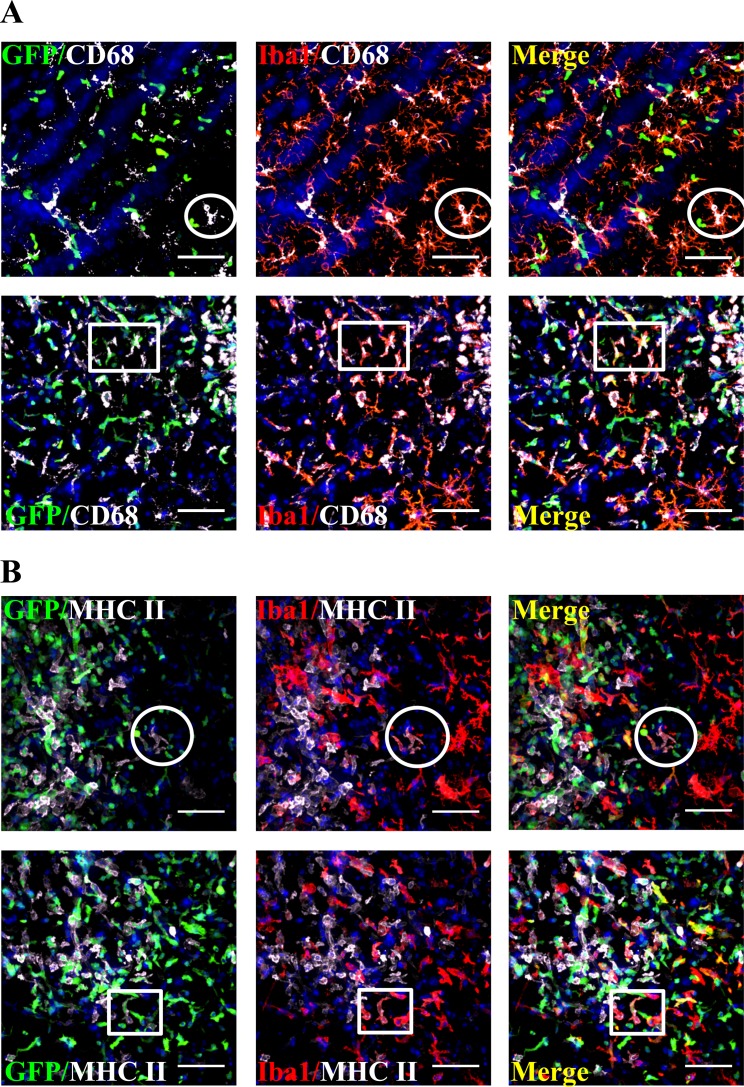
Resident microglia and monocyte-derived macrophages express the CD68 and MHC II activation markers in response to HSV-1 infection. (A) Brain sections of infected GFP^+/-^→WT mice were stained for microglial cells with rabbit anti-Iba1 and for lysosomal activation marker with rat anti-CD68 followed by secondary Alexa 594-conjugated goat anti-rabbit (red) and Cy5-conjugated goat anti-rat (white) antibodies, respectively. Nuclear staining with DAPI is shown in blue. Pictures were taken from brain sections of mice sacrificed on day 6 or 8 following infection. (B) Staining of brain sections for microglial cells with rabbit anti-Iba1 and for cell surface activation marker with rat anti-MHC II followed by secondary antibodies, Alexa 594-conjugated goat anti-rabbit (red) and Cy5-conjugated goat anti-rat (white), respectively. Both resident microglia (Iba1^+^/GFP^-^) (white circles) and blood monocyte-derived macrophages (GFP^+^/Iba1^+^) (white rectangles) expressed the lysosomal marker CD68 (Fig 6A) and the cell surface marker MHC II (Fig 6B). Scale bar 50 μm.

To investigate the involvement of resident microglia and infiltrating monocyte-derived macrophages in the immune response to HSV-1 infection, the expression of lysosomal CD68 and major histocompatibility complex (MHC) II markers, which are respectively associated with activated phagocytic macrophages and antigen-presenting cells activity [38, 41], was evaluated on brain sections. Pictures depicted in Fig 6A (CD68) and 6B (MHC II) were obtained from mice sacrificed on days 6 and 8 post-infection, which corresponded to the peak level of leukocytes infiltration. Our data showed that some GFP^-^/Iba1^+^ resident microglia displayed immuno-reactivity for both CD68 and MHC II markers at this time point (see white circles in Fig 6A and 6B, respectively) whereas the signal was barely present or not detected in non-infected mice (data not shown). Furthermore, positive signal for both markers was also observed on days 8 and 10 post-infection (data not shown). These results suggest that microglial cells were activated and could participate in antigen presentation to CD4^+^ T lymphocytes following HSV-1 infection of the CNS. Interestingly, infiltrating GFP^+^/Iba1^+^ blood-derived macrophages could express CD68 and MHC II markers (see white rectangles in Fig 6A and 6B) indicating that, once in the CNS, these cells are immuno-reactive and are also involved in the setting of the cerebral immune response during HSE.

## Discussion

In the present study, we intended to better characterize the kinetics and distribution of infiltrating blood monocytes in the CNS and to examine their immunological involvement during experimental HSE. To this aim, chimeric C57BL/6 mice were infected intranasally with a sub-lethal dose of HSV-1 sufficient to induce clinical signs of HSE (i.e., weight loss, ruffled fur, swollen forehead) without inducing mortality [19, 39]. Chemotherapy regimen consisting in the alkylating agent busulfan and the immunosuppressive drug cyclophosphamide was used to generate chimeric mice by transplantation of blood mononuclear cells that expressed the GFP from donor mice without affecting the pool of resident microglia in the CNS. Our myeloablative setting was previously shown to induce a high rate of chimerism in blood leukocytes similar to that obtained in γ-irradiated mice [36]. Importantly, in contrast to irradiation, chemotherapy prevents non-specific engraftment of transplanted cells in the brain of non-infected mice, which allows the discrimination of lesion-induced recruited cells from resident microglia [19, 36]. In our study, the evaluation of GFP-expressing cells in the blood of recipient mice showed a high proportion of cells from donor origin in the global leukocytes population. Furthermore, histologic analysis of brain sections from non-infected mice confirmed that no GFP^+^ cells could be found in the CNS parenchyma.

Flow cytometry analysis demonstrated that blood monocytes and neutrophils infiltrated the CNS of chimeric C57BL/6 mice during HSE. More precisely, our results highlighted that the profiles of infiltration of both «Ly6C^hi^» inflammatory and «Ly6C^low^» patrolling monocytes into the brain of infected mice followed different time courses with a delayed migration for patrolling monocytes. In addition, immunohistochemistry staining for infiltrating leukocytes in brain sections from infected mice indicated that these cells colonized mostly the OB and several areas of the brainstem region including the interbrain and hindbrain as well as the cerebellum, where viral particles were disseminated. Such leukocyte infiltration was observed in brains with an intact BBB as demonstrated by the absence of albumin immunostaining. Thus, in this experimental model of HSE, immune cells may traffic from the blood to the CNS in a functional manner without requiring a breakdown of the BBB. Once in the CNS, cells from the monocytic lineage (GFP^+^/Iba1^+^) had the ability to differentiate into macrophages that express the microglia marker Iba1. These cells could adopt different morphologies depending upon their differentiation and activation states. Indeed, a ramified form was observed both in early (day 4) and late (day 10) stages of HSE while an amoeboid form was mostly observed at the peak of infection (days 6 and 8), which are associated with resting and activated microglia, respectively [40, 42, 43]. Finally, our results suggest that both resident microglia and blood monocyte-derived macrophages are immunologically active in the CNS following HSV-1 infection and may be involved in antigen presentation to T lymphocytes and phagocytosis.

It is believed that monocytes migration to the CNS following cerebral insults is a hallmark of neuro-inflammation [30, 33, 34, 44]. Once infiltrated, these cells have the ability to differentiate into DCs and microglial populations [25, 32, 45, 46]. It has been brought to light that these blood-derived macrophages are able to perform effector functions including production of pro-inflammatory cytokines as well as reactive oxygen species (ROS) and nitric oxide (NO), all of which could be beneficial for microbial clearance [10, 12, 47–49]. However, uncontrolled infiltration of these cells may result in immune-induced pathology, causing disease exacerbation [7, 16, 19, 32]. Neuroinvasive infections caused by several viruses such as lymphocytic choriomeningitis virus (LCMV) [50], Theiler’s encephalomyelitis virus (TMEV) [51], tick-borne encephalitis (TBE) [52, 53] and West Nile virus (WNV) [32, 54] are associated with «Ly6C^hi^» inflammatory monocytes infiltration into the CNS, which has been demonstrated to be harmful to the host by ultimately inducing brain damages. Indeed, a blockade or a reduction of the infiltration of these cells into the CNS decreased or delayed mortality rates in different mouse models of viral infections [33, 34]. In our experimental conditions, chimeric C57BL/6 mice infected with HSV-1 did not succumb to the infection. Thus, the infiltration of «Ly6C^hi^» inflammatory monocytes into the CNS was not sufficient to induce mortality. These results suggest that these cells did not play an important role in inflammatory exacerbation in our mouse model of HSE but could be implicated in infection containment. However, it is not excluded that higher viral inoculum or the use of more susceptible mouse strains such as BALB/c mice could result in uncontrolled inflammatory monocytes infiltration which may be detrimental. In contrast, the role of «Ly6C^low^» patrolling monocytes during CNS infection is not well defined, with little prior evidences supporting their migration into inflamed CNS [30, 41]. In a mouse model of Alzheimer disease, it has been shown that patrolling monocytes are able to crawl onto the luminal cells of veins and clear vascular-located amyloid beta (Aβ) and then circulate back into the bloodstream [55]. In addition, a recent study demonstrated that this monocyte subset is recruited at later stages of the inflammatory process mediated by «Ly6C^hi^» monocytes to perform anti-inflammatory functions [26]. In our study, patrolling monocytes were recruited into the CNS at later times following infection, which corresponded to attenuated encephalitis-related clinical signs in mice, suggesting that they may have a role in the resolution phase of inflammation.

Our results showed that neutrophils exhibit a similar pattern of infiltration into the CNS as inflammatory monocytes following HSV-1 infection. These data suggest that neutrophils may be involved in the immune response in our mouse model of HSE. In susceptible BALB/c mice, it was reported that neutrophils infiltration into the CNS may be associated with disease exacerbation and death following HSV-1 infection [15, 16]. In contrast, it was shown that depletion of neutrophils in a C57BL/6 mouse model of intranasal infection did not result in disease progression suggesting that they do not seem to play a critical role in the control of infection [56]. However, this latter study did not evaluate neutrophils infiltration and virus replication in the CNS, which may vary according to virus strain. Overall, the involvement of neutrophils in the control of HSE is controversial in different studies and remains to be elucidated.

The use of chimeras with blood leukocytes expressing a GFP tracer permitted to determine the location of these cells in the CNS following HSV-1 infection. In contrast to other studies that demonstrated a restriction of cellular infiltrates to the brainstem region of C57BL/6 mice infected with HSV-1, blood leukocytes were found in the OB, the interbrain and the hindbrain in our model of HSE [7, 17, 18]. Interestingly, infiltrating leukocytes mainly migrated to anatomical regions of the CNS where the virus was detected. Despite differences in virus spread between animals, HSV-1 was mostly detected in the OB and the brainstem regions. In line with our results, a model of intranasal instillation with HSV-1 in rats demonstrated that the virus could infect neurons in the olfactory mucosa and then be transported via the glomeruli to the mitral cells within the OB. In addition, HSV-1 may enter the brain by migrating through the trigeminal nerve endings to the trigeminal ganglia to finally reach the brainstem region including the medulla oblongata and the cerebellum [57].

Several viral infections of the CNS such as WNV and human immunodeficiency virus-1 (HIV-1) were demonstrated to induce a breakdown of the BBB integrity. Indeed, some neurotropic viruses have been shown to alter the expression of junction proteins of the BBB and their functions at the multicellular interface composed of glial and vascular cells through mechanisms involving viral proteins as well as immune-mediated modulation [58, 59]. In the context of experimental HSE, it has been demonstrated that intranasal inoculation of HSV-1 to susceptible SJL mice induced the production of metalloproteinase-9 leading to the degradation of collagen IV, one of the major component of the vascular extracellular matrix scaffold. This causes a disruption of BBB integrity and an increased ability of inflammatory leukocytes to extravasate through blood vessels to infiltrate the CNS [60]. In our mouse model, blood leukocytes infiltration did not correlate with an obvious breakdown of the BBB (since no albumin signal could be detected in brain parenchyma) but might be rather related to a functional recruitment. Indeed, it has been demonstrated that a complex network between pathogens and host factors governs BBB permeability during viral infections of the CNS. Recent studies have shown that cytokines and chemokines including TNF-α, IL-1β, IFN-γ and CCL2 signal to modulate BBB function and increase its permeability to facilitate leukocytes trafficking which is associated with improved disease outcome and enhanced antiviral immunity [58].

Concurrently to infiltrating leukocytes, microglial cells play an important role during experimental HSE. In fact, it has been demonstrated that these cells are required to counter HSV-1 infection by producing cytokines such as IFN-β and IL-6 [10, 47] Furthermore, microglia were shown to increase their expression level of MHC II following HSV-1 intranasal infection in BALB/c mice as well as in a C57BL/6 mouse model of oral mucosa inoculation [15–17]. In accordance with these results, our study showed that microglia (GFP^-^/Iba1^+^) expressed MHC II and CD68 markers indicating their activation state and their eventual involvement in the immune response. Interestingly, blood-derived macrophages (GFP^+^/Iba1^+^) were also immuno-reactive for both MHC II and CD68 markers. Thus, our data argue that immune response to HSV-1 in the CNS may be elaborated by a relative contribution of both microglia and blood-derived leukocytes in our mouse model of HSE.

In conclusion, our findings demonstrated that intranasal infection of chimeric C57BL/6 mice with HSV-1 resulted in blood leucocytes infiltration to the CNS including neutrophils as well as inflammatory and patrolling monocytes. These peripheral leucocytes migrated to specific anatomical regions of the brain and could differentiate into activated macrophages that are involved, concurrently with microglia, in the cerebral immune response during HSE.
